# Traumatic Subarachnoid Hemorrhage: A Scoping Review

**DOI:** 10.1089/neu.2021.0007

**Published:** 2022-01-11

**Authors:** Dylan P. Griswold, Laura Fernandez, Andres M. Rubiano

**Affiliations:** ^1^ NIHR Global Health Research Group on Neurotrauma, University of Cambridge, Cambridge, United Kingdom.; ^2^ Division of Neurosurgery, Department of Clinical Neurosciences, Addenbrooke's Hospital and University of Cambridge, Cambridge, United Kingdom.; ^3^ Stanford School of Medicine, Stanford, California, USA.; ^4^ Neuroscience Institute, INUB-MEDITECH Research Group, El Bosque University, Bogotá, Colombia.; ^5^ Neurological Surgery Service, Vallesalud Clinic, Cali, Colombia.

**Keywords:** neurotrauma, SAH, scoping review, subarachnoid hemorrhage, TBI, traumatic brain injury

## Abstract

Sixty-nine million people have a traumatic brain injury (TBI) each year, and TBI is the most common cause of subarachnoid hemorrhage (SAH). Traumatic SAH (TSAH) has been described as an adverse prognostic factor leading to progressive neurological deterioration and increased morbidity and mortality. A limited number of studies, however, evaluate recent trends in the diagnosis and management of SAH in the context of trauma. The objective of this scoping review was to understand the extent and type of evidence concerning the diagnostic criteria and management of TSAH. This scoping review was conducted following the Joanna Briggs Institute methodology for scoping reviews. The review included adults with SAH secondary to trauma, where isolated TSAH (iTSAH) refers to the presence of SAH in the absence of any other traumatic radiographic intracranial pathology, and TSAH refers to the presence of SAH with the possibility or presence of additional traumatic radiographic intracranial pathology. Data extracted from each study included study aim, country, methodology, population characteristics, outcome measures, a summary of findings, and future directives. Thirty studies met inclusion criteria. Studies were grouped into five categories by topic: TSAH associated with mild TBI (mTBI), *n* = 13), and severe TBI (*n* = 3); clinical management and diagnosis (*n* = 9); imaging (*n* = 3); and aneurysmal TSAH (*n* = 1). Of the 30 studies, two came from a low- and middle-income country (LMIC), excluding China, nearly a high-income country. Patients with TSAH associated with mTBI have a very low risk of clinical deterioration and surgical intervention and should be treated conservatively when considering intensive care unit admission. The Helsinki and Stockholm computed tomography scoring systems, in addition to the American Injury Scale, creatinine level, age decision tree, may be valuable tools to use when predicting outcome and death.

## Introduction

It is estimated that, globally, 69 million persons (95% confidence interval [CI] 64–74 million) have a traumatic brain injury (TBI) each year.^1^ High-income countries have nearly 18 million cases, while low-and middle-income countries (LMICs) have around 50 million cases—an almost three-fold increase.^1^ This is relevant because TBI is the most common cause of subarachnoid hemorrhage (SAH). Thus, traumatic subarachnoid hemorrhage (TSAH) is a common finding in moderate and severe TBI (sTBI), because it occurs in 33–60% of patients.^2,3^ Road traffic accidents, falls, and violence are the main contributing factors to sTBI, and the majority of victims are those 15 to 44 years old in the prime of life and leading contributors to the country's gross domestic product (GDP). Thus, a country's economic security is affected by sTBI, and the country should have a vested interest in reducing its prevalence.^4^

Although it is necessary to understand this condition's pathophysiology more fully, some theories have been described in animal studies that could largely explain the clinical course of TSAH. These theories are principally concerned with the phenomenon of traumatic vasoconstriction, which contributes to secondary ischemic damage and has a variable incidence range of 19–68%. Marmarou and associates^5^ and Thomas and colleagues^6^ used a rat model to describe the significant increase of intracranial pressure (ICP) and mean arterial blood pressure changes that occur as a compensatory mechanism to maintain normal cerebral perfusion pressure.^2^

The TSAH has been described as an adverse prognostic factor leading to progressive neurological deterioration and increased morbidity and mortality. This is because of its related events of vasospasm, dyselectrolytemia, pituitary dysfunction, hypoxia, intracranial hypertension, and hydrocephalus.^3^

Current resources aim to understand the diagnosis and treatment of patients with SAH according to the severity degree of the trauma. The goal is to use this information to evaluate the cost-effectiveness of current management, reduce the length of stay (LOS), and redirect the use of already limited resources.^7^ Recently published studies have mentioned that patients with SAH secondary to mild TBI (mTBI) have a lower risk of clinical deterioration and surgical intervention,^8^ whereby the routine implementation of computed tomography (CT) scans, mandatory neurosurgery consultations, and high-intensity observations are not necessary in most cases.^7,9^

The TSAH is a public health problem of significant proportions because of the global burden of disease and its disproportionate effect on LMICs. While research has made it possible to improve the use of resource-stratified clinical interventions, it is not enough.^7^ Economies are dependent on fiscally active adults, and TSAH stunts the growth of GDP in LMICs. The implications, then, lie beyond the scope of medicine and must be taken up by economists and politicians.

Therefore, the objective of this scoping review is to develop a better understanding of TSAH. This scoping review will serve as an initial step in providing more evidence for healthcare professionals, economists, and policymakers so they might devote more resources toward this significant problem affecting both health and economic outcomes worldwide.

A preliminary search of MEDLINE, the Cochrane Database of Systematic Reviews, and Joanna Briggs Institute (JBI) Evidence Synthesis was conducted, and no current or under way systematic reviews or scoping reviews on the topic were identified.

## Methods

The proposed scoping review was conducted following the JBI methodology for scoping reviews.^10^

### Inclusion criteria

#### Participants

Studies of adult and adolescent (>15 years old) patients were included. All studies of pediatric patients (<15 years old) were excluded.

#### Concept

The concept of interest for this scoping review was studies of SAH secondary to TBI, where isolated TSAH (iTSAH) refers to the presence of SAH in the absence of any other traumatic radiographic intracranial pathology, and TSAH refers to the presence of SAH with the possibility or presence of additional traumatic radiographic intracranial pathology. Central TSAH refers to SAH present in the Sylvian fissures or subarachnoid cisterns. All studies focused on non-TSAH were excluded.

#### Context

The review was limited to studies conducted between 2005 and 2020.

### Search strategy

The search strategy aimed to locate both published and unpublished studies. An initial limited search of MEDLINE and Scopus was undertaken to identify articles on the topic. The text words in the titles and abstracts of relevant articles and the index terms used to describe the articles were used to develop a full search strategy for PubMed, MEDLINE, and Scopus. A full search strategy for MEDLINE is detailed in Appendix I. In the second phase of the search, the search strategy was adopted to search EMBASE, Web of Science, and EBSCO. The reference lists of selected studies were screened for additional studies during the third phase of the search.

Studies published in English, Spanish, and French between the years 2005 and 2020 were included.

#### Information sources

The databases searched included PubMed, Scopus, Embase, Web of Science, EBSCO, and MEDLINE.

#### Types of sources

This scoping review considered experimental and quasiexperimental study designs, including randomized controlled trials, nonrandomized controlled trials, before and after studies, and interrupted time-series studies. Analytical observational studies, including prospective and retrospective cohort studies, case-control studies, and analytical cross-sectional studies, were considered inclusion. This review also considered descriptive observational study designs, including case series, individual case reports, and descriptive cross-sectional studies for inclusion. Also, systematic reviews that met the inclusion criteria were considered. Text and opinion articles were also considered for inclusion in this scoping review.

#### Study/source of evidence selection

**FIG. 1.**
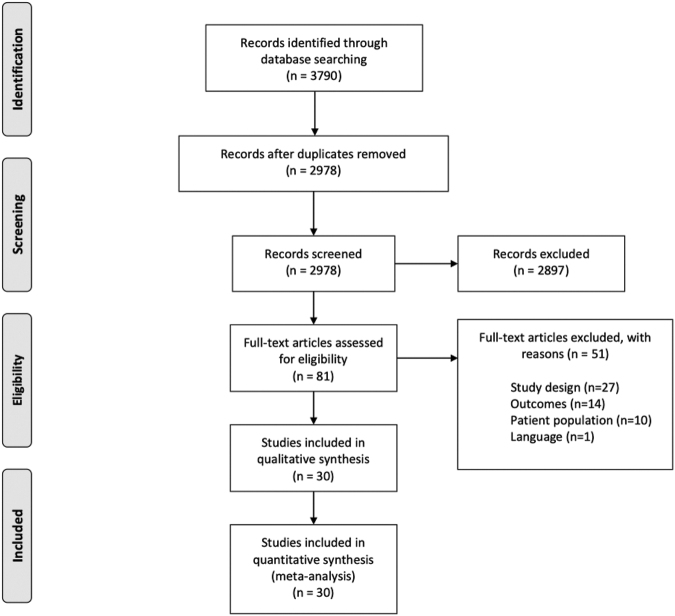
Preferred Reporting Items for Systematic Reviews and Meta-Analyses (PRISMA) flow diagram.

Following the search, all identified citations were collated and uploaded into EndNoteX9 (Clarivate Analytics, Philadelphia, PA). The citations were then imported into Covidence online software (Veritas Health Innovation, Melbourne, Australia) for screening. Two independent researchers (DG and LF) examined titles and abstracts for inclusion. The full texts of selected studies were retrieved and assessed. Full-text studies that did not meet the inclusion criteria were excluded, and the reasons for exclusion are listed in the Preferred Reporting Items for Systematic Reviews and Meta-Analyses (PRISMA) flow diagram (Fig. 1). Any disagreements that arose between the researchers during either title and abstract screening or full-text screening were resolved through discussion. Included studies underwent a process of data extraction. The results of the search are presented in the PRISMA flow diagram.

### Data

#### Data extraction

Data were extracted from the included studies by a reviewer and verified by a second reviewer using a data extraction tool developed in Covidence. The data extracted included study aim, country, methodology, length of study, sample size, population characteristics, outcome measures, a summary of findings, and conclusion and future directives. Extracted data are available in Supplementary Data 2, Table 3.

#### Data synthesis

Studies were summarized in tables, graphs, and narratively.

## Results

### Study inclusion

The study selection process is illustrated in the PRISMA flow diagram (Fig. 1.)^11^The described search identified 2978 records to screen, after which 81 studies were left for full-text review. A total of 52 articles were excluded (Supplementary Data 1). Studies were excluded based on: study design (*n* = 27), outcomes (*n* = 14), patient population (*n* = 10), and language (*n* = 1). This left 30 studies for inclusion in the final synthesis. The review included adults who had a SAH secondary to trauma, where iTSAH refers to the presence of a SAH in the absence of any other traumatic radiographic intracranial pathology, and TSAH refers to the presence of a SAH with the possibility or presence of additional traumatic radiographic intracranial pathology.

### Characteristics of included studies

Of the 30 studies, eight categories of study design were identified according to JBI methodological classification: retrospective cohort (*n* = 8), retrospective case series (*n* = 9), prospective cohort (*n* = 7), cross-sectional (*n* = 2), prospective case series (*n* = 1), systematic review (*n* = 1), meta-analysis (*n* = 1), diagnostic and test accuracy (*n* = 1). The median sample size and median study length with first and third quartile ranges were reported for each methodological category (Table 1).

**Table 1.** Study Characteristics

Study type	Count	Median sample size (IQR)	Median study length (months, IQR)
Retrospective Case Series	9	186 (63–473)	60 (36–95)
Retrospective Cohort	8	680 (452–1103)	68 (51–93)
Prospective Cohort	7	161 (129–466)	36 (36–84)
Cross–sectional	2	1130 (767–1493)	71 (47–95)
Prospective Case Series	1	7	2
Meta–analysis	1	15,372	N/A
Systematic Review	1	1074	N/A
Diagnostic and Test Accuracy	1	20	7

IQR, interquartile range.

**FIG. 2.**
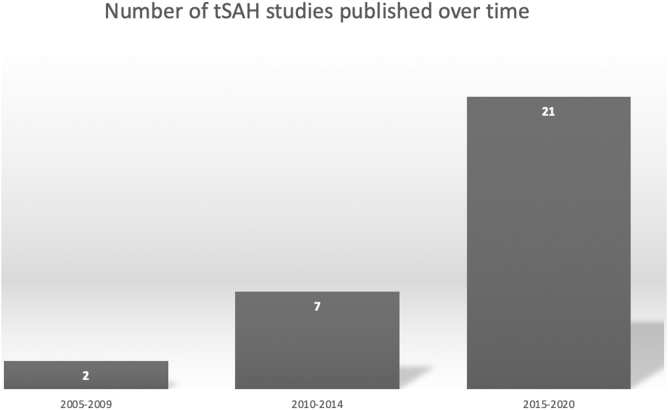
Number of relevant studies published over time. tSAH, traumatic subarachnoid hemorrhage.

Studies originated from 13 different countries (Supplementary Data 2, Table 1). According to World Bank Indexing, eight hold high-income status, two hold upper middle-income status, one holds lower middle-income status, and one (Taiwan) would hold high-income status if recognized by the United Nations as an independent member state. Two studies were published between 2005 and 2009, seven studies were published between 2010 and 2014, and 21 were published between 2015 and 2020 (Fig. 2). Studies were published in 16 different journals, 30% (*n* = 10) of which were published in the *Journal of Trauma and Acute Care Surgery*. The median impact factor (IF) was 3.3 with first and third interquartile ranges of 1.8 and 4.2, respectively (Supplementary Data 2, Table 2).

Studies were grouped into five separate categories: mTBI (*n* = 14, Table 2); sTBI (*n* = 3, Table 3); clinical management and prognosis (*n* = 9, Table 4); imaging (*n* = 3, Table 5); and traumatic aneurysm (*n* = 1, Table 6). All extraction data can be found in Supplementary Data 2, Table 3.

**Table 2.** Mild Traumatic Brain Injury

Author	Aim of study	Country	Methodology	Period	Sample size	Outcome measures	Summary of results
Levy 2011	To investigate differences between mild isolated TSAH and concussion patients. Hypothesized that mild isolated TSAH patients would not significantly differ in their clinical course or outcomes from concussion patients.	USA	Retrospective cohort	Jan 1999 – Dec 2008	1261	• ICU admission • Neurosurgical intervention • ICU LOS • Hospital LOS • Progression of TSAH • Inhospital mortality	• ICU admission: 46% (*n* = 54) • Neurosurgical intervention: 0% • Significant reduction in the odds of ICU LOS • No significant difference in hospital LOS • Mortality: 0.40%
Nassiri 2017	To review the evidence regarding patient outcomes after mTBI with iTSAH	Canada	Meta–analysis	Jan 2000 – Feb 2017	15,372	• Need for neurosurgical intervention • Radiographic progression • Neurologic deterioration • Mortality	• Neurosurgical intervention: 0.0017%. • Radiographic progression: 5.76% (n = 931) • Neurological deterioration: 0.75% (n = 10/1428) • Mortality: 0.60% (n = 5/873)
Sharma 2020	To evaluate the neurosurgical outcomes of iTSAH with GCS score of 13 to 15 who were transferred to a higher level of care.	USA	Retrospective case series	2010–2015.	11,380	• Need for neurosurgical intervention • ICU admission • ICU LOS • Hospital LOS • Mortality	• Neurosurgical intervention: 1.7% • ICU admission: 55.2% (*n* = 6280) • ICU LOS: 2 days • Hospital LOS: 3 days • Mortality: 2.2% (n = 250)
Borczuk 2013	To define which patients with mild head trauma and intracranial hemorrhage have such a low risk of deterioration that they may not need transfer for neurosurgical consultation.	USA	Cross-sectional	Jan 2009 – Dec 2010	404	• Deterioration, represented by a composite of neurosurgical intervention, clinical deterioration, or worsening CT scan result	• One patient with deterioration
Ditty 2015	To investigate the clinical implications of mTBI in an attempt to distinguish those patients who are at risk for neurological decline from those who are not	USA	Retrospective case series	May 2003 – May 2013	63	• Neurological decline • Neurosurgical intervention	• Neurological decline: 0% • Neurosurgical intervention: 0%
Gates 2017	To assess the rate of radiographic or clinical progression, the need for neurosurgical intervention, and use of health care resources, including inpatient costs, to evaluate the necessity of transfer.	USA	Retrospective cohort	Jan 2010 – Dec 2014	67	• Need for neurosurgical intervention • Radiographic progression • Clinical deterioration.	• Radiographic progression: 22.9% (*n* = 8/35) • Neurosurgical intervention: 0% • Neurological deterioration: 0%
Quigley 2013	To ascertain whether iTSAH without other intracranial pathological diagnosis is a more benign form of minor head injury that does not warrant extensive and expensive observation and follow-up	USA	Retrospective case series	2004–2011	478	• Neurological decline • Radiographic progression • Discharge status	• Radiographic progression: 3.1% (*n* = 15) • Neurological decline: 0%
Witiw 2018	To evaluate the association between hospital-level ICU admission practices and clinically important outcomes for patients with iTSAH and mTBI	Canada	Retrospective cohort	Jan 2012– Mar 2014.	14,146	• Need for neurosurgical intervention • Hospital discharge disposition • Inhospital mortality • LOS	• Neurosurgical intervention: 0.24% (*n* = 34) • Discharge home: 66.7% (*n* = 9) • LOS: 3 days
Kumar 2018	To assess the necessity of repeat head CT imaging in managing iTSAH	USA	Retrospective case series	Jan 2013 – May 2015	58	• Radiographic progression	• Radiographic progression 8.6% (*n* = 5)
Trevisi 2018	To investigate the role of repeated CT scans in the treatment of patients in neurologically stable condition who were admitted for observation after mTBI and an initially positive nonsurgical CT scan.	Italy	Retrospective case series	Jan 2008 – Dec 2015	222	• Radiographic progression • Neurosurgical intervention	• Radiographic progression: 12 h: 4 patients; 24 h: 5 patients; 48 h: 9 patients • Radiographic progression: 3 patients scanned at 12 h showed progression at 48 h • One patient on antiplatelet therapy scanned at 24 h showed progression at 48 h • Neurosurgical intervention: 0%
Diaz 2019	To demonstrate that awake patients with SAH will have lower mortality and needless interventions than awake patients with non-SAH–ICHs	USA	Retrospective cohort	Jan 2013 – Dec 2017	1051	• Need for neurosurgical intervention • Mortality	• Neurosurgical intervention: 0.89% (*n* = 2) • Mortality: 1.78% (*n* = 4/225)
Albertine 2016	To examine how the size of TSAH may impact rates of neurologic decline, medical decline, and overall patient outcome	USA	Retrospective case series	2011–2014	62	• Clinical decline • Neurosurgical intervention • ICU LOS • Hospital LOS	• Fisher grade <2, Modified Fisher <1, Claassen <1 without significant neurologic decline, medical decline, or post-hemorrhagic seizures during their hospital course • Neurosurgical intervention: 0% • LOS in ICU: 2.5 days • LOS in hospital: 4.6 days
Phelan 2014	To compare the presentation and clinical course of subjects with iTSAH to all other TBI	USA	Retrospective cohort	2010 – 2012.	698	• Radiographic progression • ICU LOS • Hospital LOS • Mortality	• Radiographic progression: 1.3% (*n* = 1) • ICU LOS: 3.1 days • Hospital LOS: 7.6 days • Mortality: 4% (*n* = 4)
Deepika 2013	To compare the impact of isolated SAH with normal CT scan on outcome of patients with mTBI	India	Retrospective case series	Jan 2010– Mar 2010	34	• Outcome (GOSE, RPCSQ score, RHFUQ score).	• No significant difference in the outcome scores between the two groups • Mean iTSAH RPCSQ: 1.38 • Mean iTSAH RHFUQ: 1.11 • Mean normal RPCSQ: 0.40 • Mean normal RHFUQ: 0.53

TSAH, traumatic subarachnoid hemorrhage; ICU, intensive care unit; LOS, length of stay; mTBI, mild traumatic brain injury; iTSAH, isolated traumatic subarachnoid hemorrhage; GCS, Glasgow Coma Scale; CT, computed tomography; SAH, subarachnoid hemorrhage; ICH, intracerebral hemorrhage; TBI, traumatic brain injury; RPCSQ, Rivermead Post–Concussion Symptoms Questionnaire; RHFUQ, Rivermead Head Injury Follow–up Questionnaire; GOSE, Extended Glasgow Outcome Scale.

**Table 3.** Severe Traumatic Brain Injury

Author	Aim of study	Country	Methodology	Period	Sample size	Outcome measures	Summary of findings
Thelin 2017	To evaluate the Stockholm and Helsinki CT scores for predicting functional outcome, in comparison with the Rotterdam CT score and Marshall CT classification. To assess which individual components of the CT scores best predict outcome and what additional prognostic value the CT scoring systems contribute to a clinical prognostic model	United Kingdom	Prospective cohort	2005–2014	1115	• CT severity score as predictor of GOS	• TSAH was the most important component of the Stockholm and Helsinki CT scoring system for outcome prediction
Matsushima 2015	To investigate the relationship between time to surgery and outcomes in patients with isolated sTBI requiring an emergent neurosurgical intervention	USA	Prospective cohort	2003 – 2013	161	• Inhospital mortality	• Inhospital mortality rate: 34.5% (early) vs. 59.1% (late) • Neurosurgical intervention was significantly associated with a higher odds of patient survival
Tu 2011	To evaluate whether the maximum thickness of subarachnoid blood is an independent prognostic marker of mortality after TSAH	China	Prospective cohort	2007–2010	104	• 1-month mortality	• Maximum thickness of subarachnoid blood is a prognostic marker of 1-month mortality

CT, computed tomography; GOS, Glasgow Outcome Score; sTBI, severe traumatic brain injuryt; TSAH: traumatic subarachnoid hemorrhage.

**Table 4.** Clinical Management and Diagnosis

Author	Aim of study	Country	Methodology	Sample size	Period	Outcome measures	Summary of findings
Wong 2011	To investigate neurological outcome among head injury patients by examining the prognostic values of CT patterns of TSAH, in particular, the thickness and distribution	China	Prospective cohort	Jan 2006–Dec 2008	661	• GOS	• Maximum thickness (mm) of SAH was independently associated with neurological outcome and death
Brelie 2015	To analyze the clinical course and identify risk factors for potential clinical and radiologic deterioration in consideration of impaired coagulation in patients with iTSAH	Germany	Retrospective case series	2003–2014	89	• Radiographical progression • Clinical deterioration • Impaired coagulation	• Radiological progression: 28.1%. • Clinical deterioration: 6.7%. • Impaired coagulation: 38% • Radiological and clinical deterioration were significantly associated with elevated INR
Chieregato 2005	To identify the factors that may predict outcomes and changes in the CT scans of lesions in a selected population of TSAH patients	Italy	Prospective cohort	Jan 1997–Jan1999	141	• GOS	• Unfavorable GOS: 19.9% (*n* = 28) • Unfavorable GOS related to age, GCS score at admission, Marshall CT score at admission, amount of subarachnoid blood, and volume of the associated contusion
Lee 2014	To provide a more comprehensive assessment of iTSAH among patients with any GCS score and to expand the analysis to examine the potential need for aggressive medical, endovascular, or open surgical interventions in these patients	USA	Retrospective cohort	Jan 2003 – Dec 2012	661	• Aggressive procedural intervention • Mortality • Hospital transfer	• Aggressive neurosurgical, medical, or endovascular intervention: 0.61% (*n* = 4) • Mortality: 1.7% (*n* = 6) • 68% of patients without additional systemic injury were discharged
Lin 2012	To investigate the impact of TSAH on outcome and clarify the role of various TSAH subgroups and to discuss the possible underlying mechanism linking certain TSAH with specific outcomes	Taiwan	Prospective cohort	3 years	117	• GOS	• Age, severity of head injury, and extensiveness of subarachnoid blood are independent predictors of poor outcome
Rau 2017 35	To construct a model for iTSAH mortality prediction using a decision tree algorithm	Taiwan	Retrospective cohort	2009–2016	545	• Mortality	• 60% of those with a head AIS >4 died • 57% of those with an AIS score ≤4, but Cr ≥1.4 and age ≥76 years died
Rau 2019	To estimate the risk of mortality in adult trauma patients with TSAH and concurrent intracranial hemorrhages compared with the risk in patients with iTSAH.	Taiwan	Cross–sectional	Jan 2009– Dec 2018	1,856	• Mortality	• iTSAH: 1.8% • SAH + one diagnosis: 7.9% • SAH + two diagnoses: 12.4% • SAH + three diagnoses: 27.3%
Vergouwen 2006	To evaluate the effect of nimodipine on outcome in patients with TSAH	Netherlands	Systematic review	Up to 2006	1,074	• GOS • Mortality	• Occurrence of poor outcome and mortality was similar in nimodipine or placebo
Mata–Mbemba 2018	To test the hypothesis that midline TSAH on initial CT may implicate the same shearing mechanism that underlies severe diffuse axonal injury (DAI)	Japan	Prospective cohort	270	Jan 2009 – Dec 2013	• GOSE	• The midline TSAH independently predicted poor GOSE score at both hospital discharge and after 6 months

CT, computed tomography; TSAH, traumatic subarachnoid hemorrhage; GOS, Glasgow Outcome Score; SAH, subarachnoid hemorrhage; iTSAH, isolated traumatic subarachnoid hemorrhage; INR, International Normalized Ratio; GCS, Glasgow Coma Scale; AIS, American Injury Scale; Cr, creatinine; GOSE, Extended Glasgow Outcome Score; DAI, diffuse axonal injury.

**Table 5.** Imaging

Author	Aim of study	Country	Methodology	Sample size	Period	Outcome measures	Summary of findings
Fotakopoulos 2018	To evaluate the diagnostic performance of different diagnostic CT scan perfusion aspects in diagnosing the clinical outcome of patients with SAH	Greece	Prospective case series	7	Jul– Sep 2016	• CBF	• CBF value of <24.5 presented 67% sensitivity and 100% specificity in diagnosis of adverse ischemic events at 1 month
Rubino 2014	To determine how often routine head CT at clinic follow-up yields clinically useful information	Lebanon	Retrospective cohort	173	Apr 2006 – Aug 2012	• Clinically relevant CT finding	• Symptomatic at follow-up and having a change in CT scan: sensitivity, 66.7%; specificity, 61.6%,
Wu 2010	To compare CT and SWI in their abilities to detect SAH and determine whether SWI can provide complementary information to CT.	China	Diagnostic and test accuracy	20	May 2008 – Dec 2008	• Radiologic identification of SAH on CT and SWI	• SWI was found to perform better than CT in detecting intraventricular hemorrhage, but worse when detecting basilar cistern SAH

CT, computed tomography; SAH, subarachnoid hemorrhage; CBF, cerebral blood flow; SWI, susceptibility weighted imaging;.

**Table 6.** Aneurysmal Traumatic Subarachnoid Hemorrhage

Author	Aim of study	Country	Methodology	Period	Sample size	Outcome measures	Summary of findings
Balinger 2015	To demonstrate that more judicious and selective use of screening CTA in the setting of TSAH should be considered	USA	Retrospective case series	2008–2012	186	• Radiological identification of aneurysm follow-up CTA	• Aneurysmal CTA: 6.99% (*n* = 13) • Ruptured aneurysm: 61.5% (*n* = 8) • Eight ruptured aneurysms (six underwent neurosurgical clipping or coiling, one underwent a ventriculostomy, and one underwent a craniotomy for evacuation of hemorrhage)

CTA, computed tomographic angiography; TSAH, traumatic subarachnoid hemorrhage.

## Review Findings

### mTBI

Studies were categorized as mTBI if the Glasgow Coma Scale (GCS) score of the patient population was 13–15 on presentation (Table 2).

A meta-analysis of 15,372 patients with TSAH found the incidence of need for eventual neurosurgical intervention to be 0.0017%.^12^ They investigators also found a 5.76% (*n* = 931) incidence for radiographical progression, 0.75% (*n* = 10/1428) incidence for neurological deterioration, and 0.60% (*n* = 5/873) incidence for death.

A retrospective cohort study of 14,146 patients with mTBI and iTSAH found the need for neurosurgical intervention to be 0.24% (*n* = 34).^13^ The discharge disposition was 66.7% (*n* = 9) to home under self-care and 8.8% (*n* = 1) to home with support. Median hospital LOS for all subjects was three days (interquartile range, 2–5 days).

A retrospective cohort study of 102 patients with iTSAH, of whom 77 had a mTBI, found that 27 underwent a routine repeat CT scan.^9^ The radiographical progression rate was 11% (*n* = 3). The other 50 patients were observed until a change occurred in their neurological examination. Of these patients, 8% (*n* = 4) needed a repeat CT scan, and 2% (*n* = 1) demonstrated radiographical progression. A total of 1.3% (*n* = 1) had clinical or radiographical hemorrhage progression. The intensive care unit (ICU) LOS was 3.1, hospital LOS was 7.6, and 4% (*n* = 4) of the patients died.

A retrospective cohort study comparing 225 patients with iTSAH and 826 patients with non-SAH-intracerebral hemorrhage (ICH) found that 0.89% (*n* = 2) patients with TSAH and 12.1% (n = 100) of patients with non-SAH-ICH required neurosurgical intervention.^14^ Mortality rates were 1.78% (*n* = 4/225) in the patients with SAH and 2.66% (*n* = 22/826) in the patients with non-SAH-ICH.

A retrospective cohort study of 117 patients with iTSAH and 1144 patients with concussion found that 46% (*n* = 54) of patients with iTSAH and 7% (*n* = 83) of patients with concussion were admitted to the ICU.^15^ No patients required neurosurgical intervention.

A retrospective cohort study of 67 patients with iTSAH found none required neurosurgical intervention.^16^ Radiographical progression was observed in eight of the 35 patients who were rescanned. The mean age in those patients was 74 years compared with 62 years in those who were not rescanned. Six of the eight patients had a slight expansion in the subarachnoid space or new intraventricular blood. One patient experienced neurological deterioration.

A retrospective cohort study of 41 patients with iTSAH found none required neurosurgical intervention,^17^ nor did any patients experience neurological worsening.

A cross-sectional study of 75 patients with iTSAH found that one 98-year-old patient experienced neurological deterioration (odds ratio [OR], 0.08) but did not require neurosurgical intervention.^8^

A retrospective case series of 11,380 patients with iTSAH requiring transfer to a higher-level facility found that 1.7% of patients required neurosurgical intervention^18^; 55.2% (*n* = 6,280) of the patients were admitted to the ICU, where their LOS was a median of two days; 62.2 % (*n* = 7077) of the patients were discharged to home without any services; 2.2% (*n* = 250) of the patients died.

A retrospective case series of 478 patients with iTSAH found that 98.3% (*n* = 470) had a second post-admission CT scan^19^; 3.1% (*n* = 15) had CT progression. No patients experienced neurological decline nor required neurosurgical intervention.

A retrospective case series of 109 patients with iTSAH found that at 12 h post first scan, radiographical progression was found in four patients.^20^ Radiographical progression was seen in five patients 24 h post first scan and in nine patients at 48 h post first scan. Three patients scanned at 12 h showed progression at 48 h. One patient on antiplatelet therapy scanned at 24 h showed progression at 48 h. No patient required neurosurgical intervention.

A retrospective case series of 58 patients with iTSAH found that radiographical progression was seen in 8.6% (*n* = 5) of patients. These patients did not require readmission. No patients required neurosurgical intervention.^21^

A retrospective case series of 62 patients with iTSAH found that patients with lower-grade TSAHs (Fisher grade <2, Modified Fisher <1, Claassen <1) did not show significant neurological decline, medical decline, or post-hemorrhagic seizures during their hospital course.^22^ Median ICU LOS was 2.5 days. No patients required neurosurgical intervention.

A retrospective case series of 34 patients with iTSAH found that compared with patients with mTBI with normal CT scans, there was no significant difference in the outcome scores between the two groups one year after injury.^23^ The telephonic Glasgow Outcome Scale-Extended (GOSE), Rivermead Head Injury Follow-up Questionnaire (RHFUQ), and Rivermead Post-Concussion Symptoms Questionnaire (RPCSQ) scores were used to assess outcome. The mean RHFUQ and RPCSQ scores for patients with isolated SAH were 1.11 and 1.38, respectively. The mean RHFUQ and RPCSQ scores for patients with normal CT scans were 0.53 and 0.40, respectively.

### sTBI

Studies were categorized as sTBI if the GCS score of the patient population was <8 on presentation.

A prospective cohort study of 1115 patients with sTBI found that TSAH was the most essential component of the Stockholm and Helsinki CT scoring system for outcome prediction. The aggregate SAH component of the Stockholm CT score was the strongest predictor of unfavorable outcome.^24^

A prospective cohort study of 161 patients with sTBI found 55.5% to have iTSAH on CT imaging. Inhospital deaths of patients whose surgery lasted less than 200 min was compared with those whose surgery lasted 200 min or longer. The inhospital mortality rate was significantly lower in the early group (34.5% vs. 59.1%, *p* = 0.03). Early neurosurgical intervention was significantly associated with higher odds of patient survival (OR, 7.41, *p* = 0.009).^25^

A prospective cohort study of 125 patients with iTSAH found that the maximum thickness of subarachnoid blood is an independent prognostic marker of one-month mortality after TSAH.^26^

### Imaging

A retrospective cohort study of 75 patients with iTSAH with at least one clinic follow-up found that the sensitivity and specificity of being symptomatic at follow-up and having a CT scan change was 66.7% and 61.6%, respectively.^27^

A diagnostic and test accuracy study of 20 patients with TSAH found that susceptibility-weighted imaging (SWI) is very sensitive to small amounts of SAH and is useful in differentiating SAH from veins.^28^ The SWI performed better than CT in detecting intraventricular hemorrhage (IVH) but worse when detecting basilar cistern SAH.

A prospective case series of seven patients with TSAH found that cerebral blood flow (CBF), as derived from CT perfusion, is a measurable index that may help detect the degree of very early cerebral ischemia in patients with SAH.^29^ A CBF value of <24.5 presented 67% sensitivity and 100% specificity in diagnosing adverse ischemic events at one month (*p* = 0.041).

### Clinical management and prognosis

A systematic review of 1074 patients with TSAH treated with nimodipine or placebo found that the occurrence of poor outcome and death was similar in both groups.^30^

A prospective cohort study of 661 patients with TSAH found that the maximum thickness (mm) of SAH was independently associated with neurological outcome (OR, 0.8) and death (OR, 1.3), but not with the extent or location of hemorrhage.^31^

A prospective cohort study of 270 patients with TSAH who underwent brain MRI within 30 days found that 28.5% (*n* = 77) had diffuse axonal injury (DAI) and that TSAH was independently associated with DAI.^32^ Midline TSAH was independently associated with both overall DAI and DAI stage two or three. The midline TSAH on initial CT had a sensitivity of 60.8%, a specificity of 81.7%, and positive and negative predictive values of 43.7% and 89.9%, respectively, for severe DAI. When adjusted for admission GCS score, the midline TSAH independently predicted low GOSE score at both hospital discharge and after six months.

A prospective cohort study of 141 patients with TSAH found that unfavorable GOS was significantly related to age, GCS score at admission, Marshall CT score at admission, amount of subarachnoid blood, and volume of the associated contusion.^33^

A prospective cohort study of 117 patients with TSAH found that age, severity of head injury, and subarachnoid blood extensiveness are independent predictors of poor outcome.^34^ Patients with extensive TSAH with IVH tend to be associated with vasospasm in the acute stage.

A retrospective cohort study of 661 patients with iTSAH found that only 0.61% (*n* = 4) patients underwent any sort of aggressive neurosurgical, medical, or endovascular intervention; 1.7% (*n* = 6) of patients died in hospital, and five of the six were older than age 80. Of patients without additional systemic injury, 68% were discharged, including 53% of patients with a GCS score 3–8. Age, severity of injury, extensive SAH, and IVH are independent predictors of poor outcome in the cohort of patients with TSAH. Patients with extensive TSAH and IVH tend to be associated with vasospasm in the acute phase.^35^

A retrospective cohort study of 546 patients with iTSAH found that of the patients with isolated TSAH, 60% of those with a head American Injury Scale (AIS) >4 died. The mortality rate was 57% for patients with an AIS score ≤4 in addition to having a creatinine level ≥1.4 mg/dL and age ≥76 years.^36^ All patients without the above criteria survived.

A retrospective case series of 89 patients with iTSAH with a GCS score >8 and a follow-up CT scan found that the radiological expansion or conversion rate of the SAH was 28.1%.^37^ The rate of clinical deterioration was 6.7%. Neither the initial pattern intracranial localization nor the number of sulci involved with the iTSAH was associated with clinical worsening. Of these patients, 38% had impaired coagulation; 17.9% of patients had elevated International Normalized Ratio (INR). Radiological and clinical deterioration was significantly associated with elevated INR.

A cross-sectional study of 1856 patients with TSAH found that patients with iTSAH had a 1.8% mortality rate, compared with patients with SAH + one diagnosis (7.9%), SAH + two diagnoses (12.4%), and SAH + three diagnoses (27.3%), where one, two, and three diagnoses indicated the existence of one, two, or three other types of intracranial hemorrhage (subdural hematoma, epidural hematoma, or ICH).^38^ When controlling for sex, age, and pre-existing comorbidities, Group II, Group III, and Group IV patients had a 4.0, 8.9, and 21.1 times higher adjusted OR for death, respectively, than the patients with iTSAH.

### Traumatic aneurysm

A retrospective case series of 186 patients with TSAH who underwent CT angiography (CTA) found that 7% (*n* = 13) of patients had an aneurysm on the follow-up CTA.^39^ Thirteen patients (6.99%) had an aneurysm on the follow-up CTA. Of those, 61.5% (*n* = 8) presented with a ruptured aneurysm. All eight patients with a ruptured aneurysm had central TSAH, defined as a SAH present in the Sylvian fissures or subarachnoid cisterns, whereas none of the five patients with an unruptured aneurysm had central TSAH. The mechanism of injury did not correlate with the presence of an aneurysm.

## Discussion

This scoping review sought to elucidate the available evidence regarding the diagnosis and management of TSAH. Ultimately, we found little evidence regarding diagnostic capability. Most studies focused on clinical prognosis, management, and outcomes. We found that the studies could be grouped into five categories: TSAH associated with mTBI; TSAH associated with sTBI; TSAH and imaging; clinical prognosis, management, and outcomes; and post-TSAH aneurysm. This discussion focuses on the evidence discovered for each of the five categories, the scoping review's limitations, and recommendations for future research.

### Study characteristics

Traumatic SAH is a topic that is rapidly increasing in interest. Only two studies on the topic were published between 2005 and 2009, with an increase to seven studies published between 2010 and 2014. From 2015 to 2020, that number has almost quadrupled, rising to 21 published studies. While this is notable, the majority of these studies come from high-income countries. Excluding China, which is set to become a high-income country within the next couple of years, only two studies are from an LMIC—Lebanon, an upper-middle income country, and India, a lower-middle income country. An estimated 90% of injury-related deaths occur in LMICs, and the resultant social and economic costs are highest for these communities.^40–42^ More research is needed to quantify the social and economic impact of the mortality rate of TSAH and its associated physical and mental disability.

Among the 30 studies, there were only two systematic reviews, one of which was a meta-analysis. The retrospective nature of most studies limits the strength of the evidence. Eight studies were retrospective case series, which inherently carries a high-level selection bias and no comparison group.

### mTBI

There were 14 studies whose patient population consisted of individuals presenting with GCS score 13–15. Overall, patients from this population were found to have very low rates of need for neurosurgical intervention. One study found that trauma patients with a GCS score of 13–15 on initial evaluation and imaging evidence of TSAH are doubtful to require neurosurgical consultation or transfer to tertiary care centers and can safely be discharged, barring the presence of other injuries or medical issues that necessitate inpatient management.^17^ Multiple other studies either found patients in this group to not require or rarely require neurosurgical management.^8,12,13,18^

Patients from this population rarely experience neurological decline, need for transfer to a higher-level facility, or undergo radiographical progression. One study found no difference in the outcome of patients with isolated SAH compared with those with normal CT scans one year after injury.^23^ One study found that the rate of neurological deterioration because of an expansion of iTSAH in patients with mTBI is low, regardless of antiplatelet and anticoagulant agents.^16^ Another study found that patients with low-grade TSAH did not demonstrate any neurological decline, medical decline, post-traumatic seizure, and most of these patients demonstrate good clinical outcomes. This suggests that patients may not require as aggressive monitoring as is currently provided for those with TSAH.^22^

The CT imaging may have little efficacy in changing mild iTSAH management and is poorly correlated with clinical progression. A less aggressive management protocol may be more appropriate for these patients.^21^Another study found that no lesions showed significant worsening on repeat imaging at 24 h or 48 h post-initial scan. Given neurological stability, a control scan can be delayed safely as long as 48 h to avoid an excessive number of unnecessary scans.^20^

Multiple studies reported high rates of admission to the ICU. One study showed that patients with TSAH patients had increased odds of admission to the ICU, yet their ICU LOS was short. This suggests that healthcare facilities should consider creating ICU criteria for the mTBI population to optimize ICU utilization. Variables such as age, comorbidities, and neurological condition could be more important indicators to consider rather than the presence of a small volume of blood in the subarachnoid space when admitting mTBI patients to the ICU.^15^ Clinical experience and acumen should be used to guide decision-making regarding ICU admission in this patient population. The presence of iTSAH, however, should not, of itself, represent an indication to admit a patient with a clinically mTBI to the critical care unit. Reevaluation of hospital-level practices may represent an opportunity for greater resource optimization.^13^

### sTBI

There is a paucity of information available for TSAH associated with sTBI. The findings were limited to three studies. One found a link between a shorter time interval to surgical intervention and improved mortality.^25^ Most studies evaluating time to treatment and SAH involve aneurysmal SAH.^43–45^ This is particularly important for patients who present to the emergency department with a low GCS score and high-risk TBI mechanism so that the diagnostic and therapeutic processes include initial resuscitation, CT scan, neurosurgery consultation, and transfer to the operating room can be expedited. A second study found that the Stockholm and Helsinki CT scores performed better than the Rotterdam and Marshall CT scores.^24^ It may be warranted to explore a move toward these new scoring systems to effectively target intensive care and surgical treatment as early as possible.

The third study showed that the maximum thickness of subarachnoid blood immediately after nonsurgical resuscitation predicted one-month mortality with 83.9% sensitivity and 67.1% specificity and that its predictive value was similar to that of the GCS score.^26^ This is important because it may imply that providers should consider the presence of TSAH and explore the predictive value of the maximum thickness of subarachnoid blood as a new independent prognostic marker of mortality. The maximum thickness of subarachnoid blood might be useful as an additional tool for risk stratification and decision-making in the acute phase of TSAH. More research into mortality, clinical and CT progression, neurosurgical intervention, and ICU course is needed.

### Imaging

We hypothesized that there would be more studies on diagnosis and imaging; however, only three studies met the inclusion criteria. We found that repeat outpatient CT of asymptomatic patients after nonoperative cerebral contusion and TSAH is unlikely to demonstrate significant new pathology. Given the cost and radiation exposure associated with CT, it may be that imaging should be reserved for patients with significant symptoms or focal findings on neurological examination. We also found that SWI may be better than CT in detecting IVH. The SWI is very sensitive to small amounts of SAH. This could be used to help differentiate SAH from veins. The SWI, however, performs poorly in detecting basilar cistern SAH. Overall, SWI may have the potential to provide complementary information to CT in imaging TSAH.

### Clinical management and prognosis

We identified nine studies that discussed the clinical management and prognosis of TSAH. We found that iTSAH, regardless of admission GCS score, is a less severe intracranial injury that is highly unlikely to require aggressive operative, medical, or endovascular intervention. It is also unlikely to be associated with significant neurological morbidity or mortality, except perhaps in elderly patients.^35^ Age, initial coma scale, extensive TSAH, and IVH are independent predictors of poor outcome in the cohort of patients with TSAH. Statistically, patients with extensive TSAH are significantly more likely to have vasospasm.^34^

We found that patients with iTSAH with impaired coagulation, especially those with elevated INR, are at risk of clinical and radiological deterioration. Despite coagulation status, routine repetition of cranial CT scan is recommended in patients with iTSAH to detect potential radiological progression, which, if detected, should result in close observation.^37^ Patients with TSAH and coexisting types of intracranial hemorrhage have a higher adjusted OR of mortality when compared with patients with an iTSAH.^36^

On imaging, the presence of midline TSAH on initial CT was associated with poor early and long-term outcomes, probably because of severe DAI. Conversely, the absence of midline TSAH on initial CT was a reliable marker to exclude severe DAI.^32^ Further, the maximum thickness of TSAH was independently associated with neurological outcome and death but not with the extent or location of hemorrhage.^31^ The amount of subarachnoid blood and associated parenchymal damage are independent factors associated with CT progression, thus linking poor outcomes and CT changes.^33^ Results do not support the finding of a beneficial effect of nimodipine on outcome in patients with TSAH. ^30^

One study established an algorithm with three components (head AIS score ≤4, Cr <1.4 mg/dL, and age <76 years) to predict death in patients with iTSAH. The algorithm may aid in identifying patients with a high risk of poor outcomes.^36^

### Traumatic aneurysm

Only one study met the inclusion criteria for post-aneurysmal TSAH. We found that the mechanism of injury does not correlate with the presence of intracranial aneurysmal disease in patients with TSAH. The presence of central TSAH and intraventricular bleeding may indicate the need for selective performance of follow-up CTA, especially in the simultaneous presence of hypertension. Patients with isolated peripheral TSAH on initial trauma workup do not seem to warrant subsequent CTA performance.

It is important to note here this study's inability to establish cause-and-effect relationships. It is impossible to rule out whether the traumatic incident (MVA, fall) was preceded by the ruptured aneurysm. Further, while the study states that of the five of 13 unruptured intracranial aneurysms were “deemed to be proximally unrelated to the trauma and did not require immediate intervention,” it does not provide the method by which this was determined. This explanation further obfuscates the study's definition of traumatic aneurysm, because the method for determining the certainty with which the ruptured aneurysm was associated with the traumatic incident is also left out.

Last, a high parietal SAH with head trauma is less likely to indicate SAH from a ruptured aneurysm compared with a large SAH in the basal cisterns. There is a need for additional vascular studies to better characterize the nature of traumatic aneurysms. Therefore, the results of this study should be interpreted with a high risk of bias.

## Conclusions

The TSAH is a public health problem of significant proportions because of the global burden of disease and its disproportionate effect on LMICs. We found that patients with TSAH associated with mTBI have a lower risk of clinical deterioration and surgical intervention. The routine implementation of CT scans, mandatory neurosurgery consultations, and high-intensity observations are not necessary in most cases. Imaging is especially critical for TSAH associated with sTBI. The Stockholm and Helsinki scores may offer improvements in outcome predictions to target intensive care and surgical treatment early on in the course of treatment. The decision tree that evaluates head AIS, Cr, and age may help identify patients with a higher risk of death in conjunction with imaging. The presence of central TSAH and intraventricular bleeding may warrant CTA to rule out post-traumatic aneurysmal SAH.

The principal limitation of this and any scoping review is the quality of the included studies. We did not use a tool to assess the quality of the included literature because this is not characteristically a component of scoping review methodology. There were no randomized controlled trials, leaving the included studies with a higher risk of bias. There are invariably other ways to group the included studies. We arranged the studies, however, into five groups based on our best understanding of the evidence.

### Recommendations for Research

The evidence on TSAH and mTBI greatly outweighs that which is available for TSAH and sTBI. More research is needed for mortality, CT progression, neurosurgical intervention, and ICU course. Further, more research is needed for diagnostic imaging. With advances in imaging occurring at a high rate, patients need to receive optimal care, for which diagnostic capability is a priority. More research is needed to uncover the role of vasospasm in TSAH, because it is a common sequela that is improperly understood.

## Supplementary Material

Supplemental data

Supplemental data

## Supplementary Material

Supplementary Review

## Appendix

**Appendix I:** Search Strategy

MEDLINE	Query	Records retrieved
#1	((“injuries”[MeSH Subheading] OR “injuries”[All Fields] OR “trauma”[All Fields] OR “wounds and injuries”[MeSH Terms] OR (“wounds”[All Fields] AND “injuries”[All Fields]) OR “wounds and injuries”[All Fields] OR “trauma s”[All Fields] OR “traumas”[All Fields]) AND (“subarachnoid haemorrhage”[All Fields] OR “subarachnoid hemorrhage”[MeSH Terms] OR (“subarachnoid”[All Fields] AND “hemorrhage”[All Fields]) OR “subarachnoid hemorrhage”[All Fields]) AND (“diagnosable”[All Fields] OR “diagnosi”[All Fields] OR “diagnosis”[MeSH Terms] OR “diagnosis”[All Fields] OR “diagnose”[All Fields] OR “diagnosed”[All Fields] OR “diagnoses”[All Fields] OR “diagnosing”[All Fields] OR “diagnosis”[MeSH Subheading])) OR ((“injuries”[MeSH Subheading] OR “injuries”[All Fields] OR “trauma”[All Fields] OR “wounds and injuries”[MeSH Terms] OR (“wounds”[All Fields] AND “injuries”[All Fields]) OR “wounds and injuries”[All Fields] OR “trauma s”[All Fields] OR “traumas”[All Fields]) AND (“subarachnoid haemorrhage”[All Fields] OR “subarachnoid hemorrhage”[MeSH Terms] OR (“subarachnoid”[All Fields] AND “hemorrhage”[All Fields]) OR “subarachnoid hemorrhage”[All Fields]) AND (“diagnosable”[All Fields] OR “diagnosi”[All Fields] OR “diagnosis”[MeSH Terms] OR “diagnosis”[All Fields] OR “diagnose”[All Fields] OR “diagnosed”[All Fields] OR “diagnoses”[All Fields] OR “diagnosing”[All Fields] OR “diagnosis”[MeSH Subheading]))	3430
Filters	Humans, English, French, Spanish from 2005–2020	1678
